# Long-term outcome of patients with atrial fibrillation and heart failure with preserved ejection fraction after combined radiofrequency catheter ablation and left atrial appendage closure

**DOI:** 10.3389/fcvm.2026.1871243

**Published:** 2026-06-22

**Authors:** Qian-ji Che, Yi-Chao Zhang, Mu Chen, Peng-cheng Yao, Qun-Shan Wang, Jian Sun, Wei Li, Bo Liu, Peng-Pai Zhang, Yi-Chi Yu, Yu-li Yang, Mei Yang, Rui Zhang, Yi-Gang Li

**Affiliations:** Department of Cardiology, Xinhua Hospital, School of Medicine, Shanghai Jiao Tong University, Shanghai, China

**Keywords:** atrial fibrillation, heart failure with preserved ejection fraction, left atrial appendage closure, radiofrequency catheter ablation, stroke prevention

## Abstract

**Background:**

In patients with heart failure with preserved ejection fraction (HFpEF), further investigation of the safety and long-term efficacy of combined radiofrequency catheter ablation and left atrial appendage closure (RF + LAAC) in patients with HFpEF is warranted.

**Methods:**

In this retrospective study, we aimed to evaluate the outcome of RF + LAAC in patients with AF and HFpEF, compared to radiofrequency catheter ablation (RF) alone. 331 patients with AF and chronic HFpEF were divided into two groups: RF + LAAC (*n* = 196) and RF (*n* = 135). After 1:1 propensity score matching (PSM), 96 matched pairs were enrolled in further analysis. HF rehospitalization, ischemic stroke/transient ischemic attack (TIA), and major bleeding events were assessed over 24 months. Kaplan–Meier analyses were used to compare outcomes.

**Results:**

The RF + LAAC group demonstrated comparable procedural complication rates to RF alone (5.2% vs. 4.2%, *p* = 0.733). The risks of ischemic stroke/TIA (ARR=4.95%, HR = 0.096, 95%CI:0.012–0.753, *p* = 0.026) and major bleeding events (ARR=3.91%, HR = 0.120, 95%CI: 0.015–0.957, *p* = 0.045) were significantly lower in RF + LAAC group vs. RF alone, while HF rehospitalization was similar between the two groups (ARR=−0.56%, HR = 1.356, 95%CI: 0.303–6.059, *p* = 0.690).

**Conclusions:**

In patients with AF and HFpEF, combined RF + LAAC was associated with lower risks of ischemic stroke/TIA and major bleeding, without an observed increase in HF rehospitalization, compared with RF alone.

## 1 Introduction

Atrial fibrillation (AF) and heart failure with preserved ejection fraction (HFpEF) are two widely prevalent and closely associated conditions. Clinical/subclinical HFpEF coexists in 73% of the AF population, and 40%–60% patients diagnosed with HFpEF are complicated with AF (1, 2). Common risk factors such as aging, obesity and hyperglycemia are shared by both diseases, while compared with patients having either AF or HFpEF, worse clinical outcomes have been observed in patients with both diseases, both face increased risk of stroke (3–5).

It is already demonstrated that the combined procedure of radiofrequency catheter ablation and left atrial appendage closure (RF + LAAC) is a safe and effective option to attain rhythm control and stroke prevention simultaneously, even in HF patients with reduced left ventricular ejection fraction (LVEF) (6, 7). However, these findings have not yet been adequately evaluated in patients with HFpEF. In this study, we investigated the procedural safety and long-term effectiveness of RF + LAAC in patients with AF and HFpEF in comparison with AF and HFpEF patients who only received radiofrequency catheter ablation (RF).

## 2 Methods

### 2.1 Study population

In this retrospective cohort study, AF patients who underwent successful RF + LAAC between July 2019 and July 2022 and were enrolled in the LAACablation registry were included. The LAACablation registry (ClinicalTrials.gov ID: NCT03788941) is an observational single-center cohort study recruiting patients who underwent successful RF + LAAC procedures. On the other hand, the RF group was recruited by reviewing medical records and follow-up data of AF patients who only received successful RF in the same center, Xinhua Hospital, Department of Cardiology during the same period. HFpEF was identified according to the diagnostic criteria in 2021 ESC Guidelines for the diagnosis and treatment of acute and chronic HF, based on clinical manifestation, ultrasound indices and serum NT-proBNP (8).

Patients with any one of the following criteria were excluded: (1) age<18; (2) having history of hospitalization for acute HF exacerbation in the past three months; (3) valvular heart disease or ischemic cardiomyopathy; (4) abnormal thyroid function; (5) CHA₂DS₂-VASc score ≤ 1(male) or ≤ 2 (female); (6) history of LAAC.

A total of 358 patients were then enrolled in the further research. All patients underwent electrocardiography (ECG) monitoring using bedside monitors or portable monitoring devices upon admission. Additionally, routine Holter monitoring was performed to confirm the diagnosis and classification of AF (paroxysmal, persistent, or long-standing persistent) as defined in prior studies (9).

**Figure 1:**
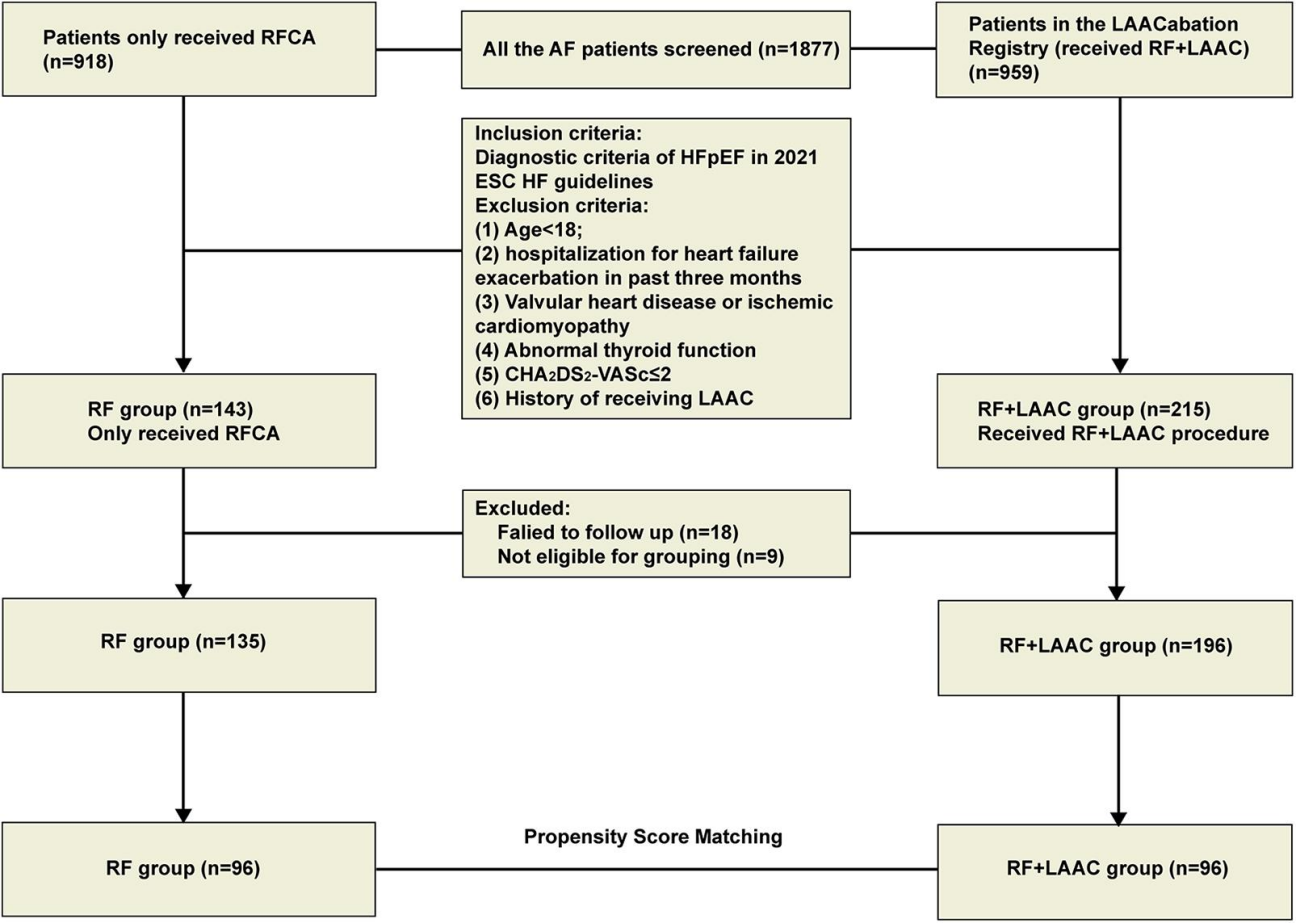
Study design. AF, atrial fibrillation; RF, radiofrequency catheter ablation; RF + LAAC, combined radiofrequency catheter ablation and left atrial appendage closure; HFpEF, heart failure with preserved ejection fraction; HF, heart failure; ESC, European Society of Cardiology; CHA2DS2-VASc, Congestive Heart Failure, Hypertension, Age ≥ 75 [Doubled], Diabetes Mellitus, prior stroke or transient ischemic attack [Doubled], Vascular Disease, Age 65–74, Female; LAAC, left atrial appendage.

After excluding 18 patients (5.4%) who were lost to follow-up and 9(2.7%) patients who did not meet the grouping criteria, a total of 331 patients were included in the final analysis. Patients in the RF group never underwent LAAC and were only given standardized drug therapies during the follow-up period (Figure 1). This study was approved by the ethics board of Xinhua Hospital and complied with the Declaration of Helsinki Principles (Approval No. XHEC-D-2025-056).

### 2.2 Treatment

#### 2.2.1 Medication

All patients received guideline-directed medical therapy (GDMT) for HF after admission to ensure surgical tolerance (8, 10, 11). The medication was dynamically adjusted based on each patient's condition at discharge and during follow-up. Additionally, all patients received standardized anticoagulation therapy after assessing embolism risk using the CHA₂DS₂-VASc score and bleeding risk using the HAS-BLED score, respectively. Antiplatelet agents were also prescribed when clinically indicated.

#### 2.2.2 Radiofrequency catheter ablation

The CARTO navigation system (Biosense Webster Inc., Diamond Bar, CA, USA) was used for atrial reconstruction and guidance of AF ablation. The procedures were uniformly conducted under conscious sedation. Three catheters were introduced: (1) a steerable coronary sinus (CS) catheter (2–5-2 mm, Dynamic XT; Boston Scientific, Marlborough, MA, USA); (2) the THERMOCOOL SMARTTOUCH SF (STSF) catheter (Biosense Webster, Inc.) with a 56-hole irrigation tip; and (3) a multipolar mapping catheter (Pentaray; Biosense Webster Inc.).

All patients received a tailored catheter ablation. For patients with paroxysmal AF, pulmonary vein isolation (PVI) was routinely delivered, while in persistent AF patients, a ’stepwise strategy’ was generally followed (12). The procedural end point was successful PVI and bidirectional block of the ablation lines, validated through differential pacing techniques and activation mapping after restoring sinus rhythm.

#### 2.2.3 Left atrial appendage closure

LAAC with WATCHMAN 2.5 (Boston Scientific, Natick, MA, USA) device was subsequently performed after the ablation procedure under the guidance of fluoroscopy. The procedure was also performed through a transfemoral approach under conscious sedation, and device size was selected according to the LAA angiography. The deployed device was required to meet the PASS criteria (Position-Anchoring-Size-Seal) (13). Immediate intra-procedural TEE and/or angiography were further performed to re-verify the appropriate implantation of the device.

### 2.3 Post-procedure management and follow-up

Vital signs and post-operative complications including cardiac tamponade, pericardial effusion that not requiring pericardiocentesis, device embolism, stroke/TIA/systemic embolism, air embolism, major bleeding and death were routinely monitored post-operatively.

Standardized antithrombotic therapies were administered in all patients at discharge (8, 10, 11). In the RF + LAAC group, patients received oral anticoagulation [warfarin or direct oral anticoagulants (DOACs)] for the first six months postoperatively if no contraindications were present. At the six-month follow-up, if no >5 mm peri-device leaks (PDLs) or device-related thrombi (DRT) were detected, anticoagulants were switched to antiplatelet drugs (13).

The first three months post-procedure were considered a “blanking period”, during which patients continued taking rhythm-control medications (amiodarone, propafenone, or sotalol) if needed. AF recurrence was defined as the occurrence of atrial fibrillation, atrial flutter, or atrial tachycardia lasting more than 30 s after the 3-month blanking period.

All patients were scheduled for outpatient follow-ups at 3, 6, 9, 12 and 24 months after discharge. Home visits, telephone follow-ups, and electronic medical record reviews were also used as supplementary methods. At 6 months after discharge, transesophageal echocardiography (TEE) or computed tomography angiography (CTA) was performed in the RF + LAAC group to check for PDLs or DRT. At each follow-up, patients underwent 12-lead ECG, Holter, with medications adjusted as needed.

The outcome events of interest in this study included HF rehospitalization, ischemic stroke/TIA, and non-operative major bleeding events defined as type 3, 4 or 5 according to the Bleeding Academic Research Consortium (BARC≥3) criteria (14, 15). Ischemic stroke/TIA was adjudicated through medical record reviews (inpatient, outpatient, and emergency visits due to ischemic stroke/TIA) and telephone follow-ups, and the diagnosis was supported by imaging evidence and medical records from neurologists.

### 2.4 Statistics

Baseline characteristics, laboratory findings, and treatment variables were summarized using descriptive statistics. Continuous variables were presented as mean ± standard deviation (SD) or as median with the 25th and 75th percentiles (Q1, Q3), while categorical variables were expressed as counts and percentages. For comparisons between groups, continuous variables were analyzed using a two-tailed t-test. Categorical variables were compared using either the Pearson chi-square test or Fisher's exact test, depending on data distribution characteristics. Time-to-event outcomes were analyzed using Kaplan–Meier curves and Cox proportional hazards models. Considering the non-randomized design, propensity score matching (PSM) was performed via logistic regression model. A rigorous nearest-neighbor matching algorithm without replacement was attempted, using a caliper width of ≤0.01. Standardized biases were calculated before and after PSM, among which a value of < 0.2 was considered the indicator of adequate bias reduction. Missing baseline data were handled using multiple imputation before PSM. All statistical analyses were performed using R 4.3.3 for data processing and visualization, ensuring analytical accuracy and reproducibility.

## 3 Results

### 3.1 Patient demographics

A total of 331 patients (female: 44.7%, average age: 71.5y) were included in the final study cohort with two groups divided: RF + LAAC group (*n* = 196),RF group (*n* = 135). After a PSM process using all baseline variables listed in Table 1, 96 pairs were enrolled in further analysis (Figure 1). All these baseline characteristics before and after matching were summarized and analyzed in Table 1. After statistical testing, a standardized bias of < 0.20 was observed in all covariates after matching, indicating no significant difference was observed on baseline characteristics between the two matched groups.

**Table 1:** Baseline characteristics.

Baseline characteristics	Before matching		After matching		Standard bias	
	RF + LAAC group (*n* = 196)	RF group (*n* = 135)	RF + LAAC group (*n* = 96)	RF group (*n* = 96)	Before matching	After matching
Female	92 (46.9)	56 (41.5)	45 (46.9)	44 (45.8)	0.11	0.02
Age	72.9 ± 6.5	68.2 ± 9.6	71.8 ± 6.5	71.2 ± 7.2	0.57	0.10
BMI	24.8 ± 3.4	26.5 ± 4.6	25.3 ± 2.8	25.1 ± 3.5	0.41	0.04
pAF	52 (26.5)	42 (31.1)	29 (30.2)	25 (26.0)	0.10	0.11
peAF	144 (73.5)	92 (68.1)	67 (69.8)	71 (74.0)	0.12	0.11
lpeAF	0	1 (0.7)	0	0	0.13	0.00
Hypertension	165 (84.2)	109 (80.7)	79 (82.3)	83 (86.5)	0.09	0.10
CAD	87 (44.4)	60 (44.4)	43 (44.8)	47 (49.0)	0.00	0.08
Diabetes	68 (34.7)	37 (27.4)	27 (28.1)	28 (29.2)	0.16	0.00
CI/TIA	52 (26.5)	40 (29.6)	24 (25.0)	23 (24.0)	0.07	0.00
HCM	5 (2.6)	6 (4.4)	4 (4.2)	1 (1.0)	0.11	0.19
DCM	0	2 (1.5)	0	0	0.19	0.00
COPD	4 (2.0)	3 (2.2)	1 (1.0)	2 (2.1)	0.01	0.09
NYHA II	142 (72.4)	107 (79.3)	74 (77.1)	75 (78.1)	0.16	0.00
NYHA III	54 (27.6)	28 (20.7)	22 (22.9)	21 (21.9)	0.16	0.00
CHA2DS2-VASc score	4.8 ± 1.4	4.3 ± 1.2	4.6 ± 1.1	4.6 ± 1.2	0.38	0.05
Major bleeding history	6 (3.1)	3 (2.2)	3 (3.1)	1 (1.0)	0.05	0.14
HAS-BLED score	2.0 ± 0.7	1.9 ± 0.7	1.9 ± 0.7	2.0 ± 0.6	0.23	0.03
Implanted PM	7 (3.6)	9 (6.7)	7 (7.3)	6 (6.3)	0.14	0.03
RF history	18 (9.2)	12 (8.9)	9 (9.4)	10 (10.4)	0.01	0.02
LVEF	62.0 ± 4.6	61.5 ± 5.0	61.9 ± 4.6	61.7 ± 4.8	0.10	0.04
LAD	44.0 ± 5.2	45.0 ± 6.0	44.1 ± 5.7	44.3 ± 6.0	0.17	0.02
PASP	36.2 ± 8.6	37.5 ± 9.0	37.3 ± 9.1	36.5 ± 6.7	0.16	0.10
E/e’ ratio	18.2 ± 6.1	18.3 ± 5.9	18.2 ± 6.4	18.0 ± 5.8	0.00	0.03
H2F-PEF score	6.5 ± 0.7	6.4 ± 0.6	6.4 ± 0.6	6.4 ± 0.7	0.14	0.02
NT-proBNP	1,119.0 (800.4, 1,779.0)	798.9 (477.5,1,563.8)	1,080.5 (766.6,1,505.5)	793.9 (460.7,1,523.3)	0.08	0.12
cTnI	0.01 (0.005,0.02)	0.01 (0.005,0.02)	0.01 (0.005,0.02)	0.01 (0.005,0.02)	0.02	0.09
Hb	135.1 ± 18.4	139.9 ± 18.4	137.0 ± 19.1	137.1 ± 18.5	0.26	0.00
Cr	75.0 (59.5,89.5)	79.0(61.0,93.0)	71(60.0,87.4)	78(60.8,93.0)	0.05	0.06

BMI, body mass index; HAS-BLED score, hypertension, abnormal renal and/or liver function, previous stroke, bleeding history or predisposition, labile international normalized ratios, elderly, and concomitant drugs and/or alcohol excess; H2F-PEF score, heavy (BMI>30 kg/m2)[doubled], hypertensive, atrial fibrillation [tripled], pulmonary hypertension, elder (age>60), filling pressure (E/e’>9); COPD, chronic obstructive pulmonary disease; TIA, transient ischemic attack; LAD, left atrial diameter; PASP, pulmonary artery systolic pressure; NT-proBNP, N-terminal-proB-type natriuretic peptide; cTnI, Troponin I.

NT-proBNP, cTnI and Cr were presented as median with the 25th and 75th percentiles (Q1, Q3).

### 3.2 Treatment

#### 3.2.1 Procedural characteristics and complications

Regarding ablation procedure, all patients underwent pulmonary vein isolation (PVI). Details of procedural characteristics were listed in Table 2. Both total procedure time and fluoroscopy time were significantly longer in the RF + LAAC group. No other significant difference was observed in ablation procedures between the two groups. Because the LAACablation registry only enrolled patients who underwent successful LAAC implantation, procedural success was 100% by design.

**Table 2:** Procedural characteristics and complications.

Variable	RF + LAAC (*n* = 96)	RF (*n* = 96)	*p* value
Procedural characteristics			
PVI	96 (100.0)	96 (100.0)	1.000
LA roof line	62 (64.6)	64 (66.7)	0.761
Mitral isthmus line	38 (39.6)	43 (44.8)	0.465
CFAE ablation	60 (62.5)	58 (60.4)	0.767
Cavo-tricuspid line	40 (41.7)	39 (40.6)	0.883
LA posterior lines	22 (22.9)	22 (22.9)	1.000
LA inferior lines	8 (8.3)	4 (4.2)	0.233
Anterior septal line	11 (11.5)	7 (7.3)	0.322
VOM ethanol infusion	11 (11.5)	16 (16.7)	0.299
Intracardiac cardioversion	43 (44.8)	50 (52.1)	0.312
Intraprocedural sinus rhythm restoration	53 (55.2)	46 (47.9)	0.312
Total procedure time(min)	183.5 (39.7)	164.6 (38.7)	0.001
Fluoroscopy time(s)	748.7 (293.4)	498.4 (173.7)	0.000
LAA diameter	22.6 (3.5)	/	/
Device diameter	27.6 (3.7)	/	/
Procedural complications			
Total	5 (5.2)	4 (4.2)	0.733
Cardiac tamponade	2 (2.1)	0	0.155
Pericardial effusion not requiring pericardiocentesis	3 (3.1)	3 (3.1)	1.000
Device embolism	0	0	1.000
Stroke/TIA/systemic embolism	0	1 (1.0)	0.316
Air embolism	0	0	1.000
Major bleeding	0	0	1.000
Death	0	0	1.000

PVI, pulmonary vein isolation; CFAE, complex fractionated atrial electrogram; VOM, vein of Marshall; LAA, left atrial appendage.

The overall complication rates in both groups were comparable as shown in Table 2. In the RF + LAAC group, 2 patients (2.1%) encountered pericardial tamponade; 3 patients (3.1%) developed mild to moderate pericardial effusion that did not require pericardiocentesis. In the RF group, no patient encountered pericardial tamponade; 3 patients (3.1%) developed mild to moderate pericardial effusion that did not require pericardiocentesis. Additionally, 1 patient (1.0%) in the RF group experienced ischemic stroke within 24 h after procedure. No device embolism, major bleeding, or death were observed in either group. Incidence rate of various types of complications were comparable between the two groups (Table 2).

#### 3.2.2 Medication

All of the 96 patients (100%) in the RF + LAAC group were prescribed DOACs at discharge, while 96.9% patients (*n* = 93) were using DOACs, and the remaining 3.1% (*n* = 3) were using warfarin. Then all patients in the RF + LAAC group routinely switched from oral-anticoagulant drugs to antiplatelet drugs at 6 months post-procedure, and both RF + LAAC and RF groups discontinued all AADs at the end of 3-month blanking period. To reflect the long-term medication use in each group, a summary of medication use at the 12-month follow-up was demonstrated (Table 3).

**Table 3:** Medication characteristics.

Medication	Antithrombotics at discharge		
	RF + LAAC (*n* = 96)	RF (*n* = 96)	*p* value
Warfarin	0	3 (3.1)	0.081
DOAC	96 (100.0)	93 (96.9)	0.081
Antiplatelets	0	0	1.000

	Prescription at 12-month follow-up		
	RF + LAAC (*n* = 95)*	RF (*n* = 96)	*p* value
Antithrombotics			
Warfarin	0	3 (3.1)	0.082
DOAC	0	92 (95.8)	<0.001
Antiplatelets	95 (100.0)	3 (3.1)	<0.001
Antiarrhymic drugs			
Class I *	0	0	1.000
Amiodarone	8 (8.4)	13 (13.5)	0.258
Other Class III	4 (4.2)	3 (3.1)	0.690
Class IV	4 (4.2)	3 (3.1)	0.690
Beta-blocker	24 (25.3)	20 (20.8)	0.467
Anti-HF drugs			
ACEi/ARB/ARNi	43 (45.3)	40 (41.7)	0.616
MRA	4 (4.2)	8 (8.3)	0.240
SGLT2i	3 (3.2)	4 (4.2)	0.711
Diuretics	20 (21.1)	19 (19.8)	0.829
Digitalis	0	1(1.0)	0.319

DOAC, direct oral anticoagulants; ACEi, angiotensin-converting enzyme inhibitor; ARNi, angiotensin receptor-neprilysin inhibitor; ARB, angiotensin II receptor blocker; MRA, mineralocorticoid receptor antagonist; SGLT2i, sodium-glucose co-transporter 2 inhibitor.

*1 patient in the RF + LAAC group died at the 8th month after procedure.

All patients in the RF + LAAC group were routinely switched to antiplatelet drugs. In the RF group, 3(3.1%) patients used warfarin; 92(95.8%) patients used DOACs; and 3(3.1%) patients used antiplatelet drugs. Due to atrial fibrillation recurrence, several patients in the RF + LAAC and RF groups were still using AADs at one year postoperatively. Except for significant difference in the use of DOACs and warfarin as designed, the use of other drugs were similar across the two groups.

### 3.3 Outcomes

In the 6-month follow-up when every patient received TEE or CTA in the RF + LAAC group, a severe PDL (≥5 mm) was found in 3 patients (3.1%), while DRT was detected in 1 patient (1.0%).

**Figure 2:**
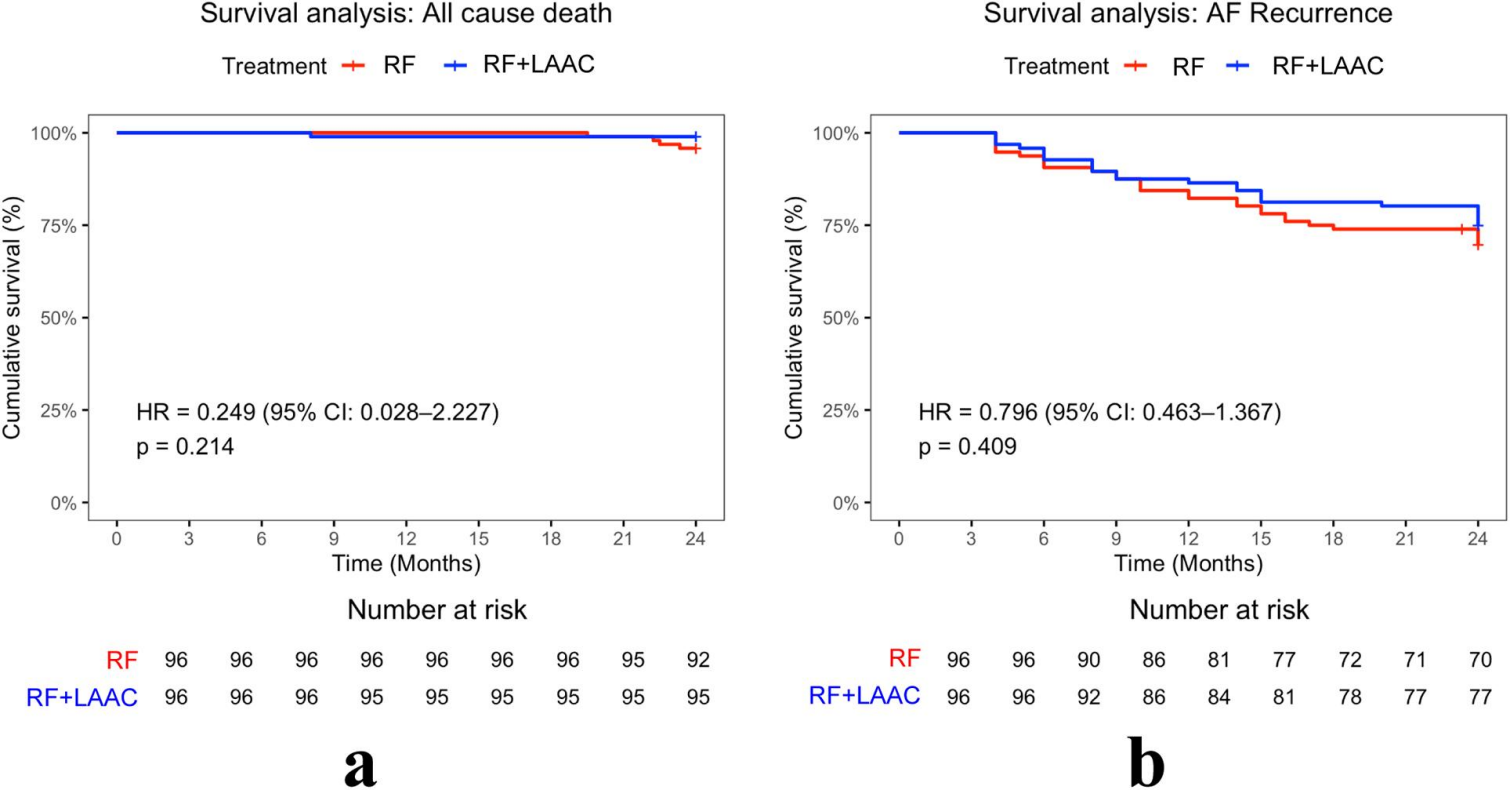
Summary of death and AF recurrence during follow-up period **(a)** cumulative incidence of all cause death. **(b)** Cumulative incidence of atrial fibrillation recurrence.

During the 24-month follow-up period, the all-cause death rate per 100 patient-years in the RF + LAAC group was 0.52 (*n* = 1), and the number was 2.09 (*n* = 4) in the RF group (HR = 0.249, 95% CI: 0.028–2.227, *p* = 0.214). In terms of AF recurrence after procedure, a total of 24 patients (rate per 100 patient-years: 14.19) in the RF + LAAC group experienced atrial fibrillation recurrence, while 29 patients (rate per 100 patient-years: 17.83) in the RF group encountered atrial fibrillation recurrence (HR = 0.796, 95% CI: 0.463–1.367, *p* = 0.409). Cox analysis revealed no significant difference between the two groups (Figure 2).

**Figure 3:**
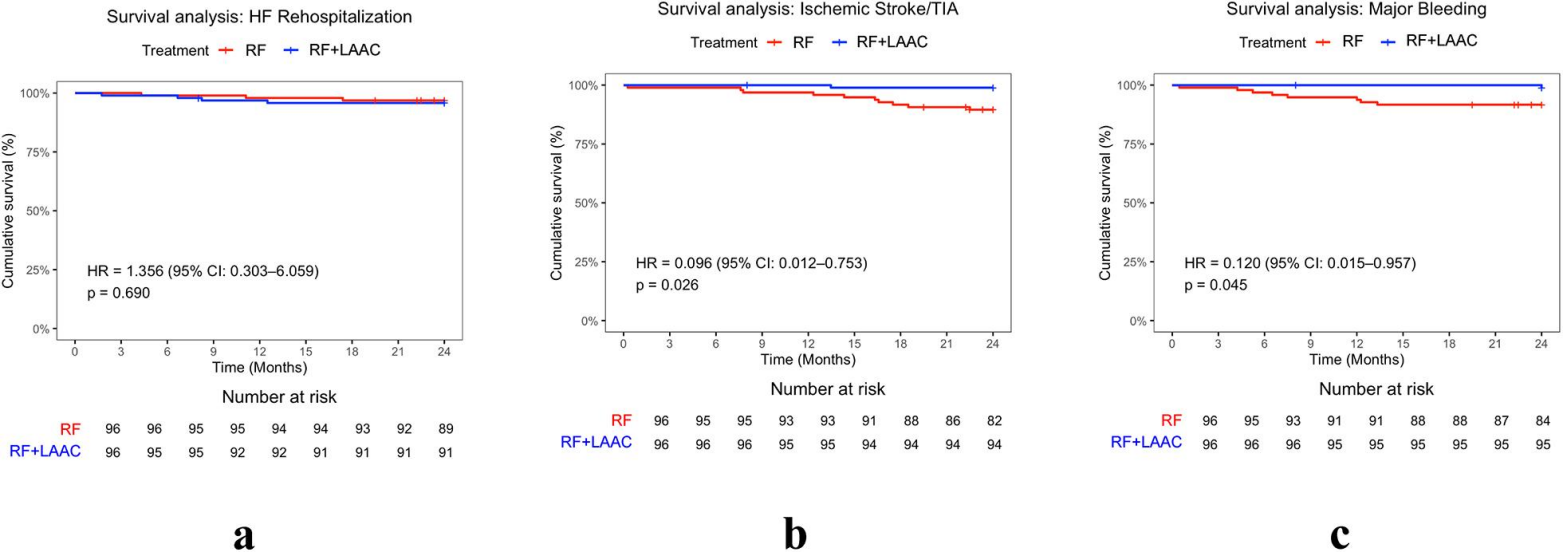
Kaplan–meier analysis of endpoint events. **(a)** Cumulative incidence of heart failure rehospitalization events. **(b)** Cumulative incidence of stroke/TIA events. **(c)** Cumulative incidence of major bleeding events. TIA, transient ischemic attack.

4 patients (rate per 100 patient-years: 2.16) in the RF + LAAC group experienced HF rehospitalization recurrence during the 24-month follow-up, while the number was 3 (rate per 100 patient-years: 1.60) for the RF group. Ischemic stroke/TIA was recorded in 10 patients in the RF group (rate per 100 patient-years: 5.48), and the figure was 1 in the RF + LAAC group (rate per 100 patient-years: 0.53). The rate of major bleeding was 4.43 per 100 patient-years in the RF group (*n* = 8) and 0.52 in the RF + LAAC group (*n* = 1). Survival data were compared using the Log-Rank test. Compared to the RF group, similar risk of HF rehospitalization was also observed (ARR=−0.56%, HR = 1.356, 95% CI: 0.303–6.059, *p* = 0.690). However, the RF + LAAC group witnessed a significantly reduced occurrence of ischemic stroke/TIA compared to the RF group (ARR=4.95%, HR = 0.096, 95% CI: 0.012–0.753, *p* = 0.026). A significant reduction in major bleeding events were also observed in the RF + LAAC group, compared to the RF group (ARR=3.91%, HR = 0.120, 95% CI: 0.015–0.957, *p* = 0.045). The results were all demonstrated in Figure 3.

To evaluate the robustness of the matched analyses, Firth-penalized Cox proportional hazards regression was performed adjusting for age, sex, CHA₂DS₂-VASc score, HAS-BLED score, LVEF, LAD, and NT-proBNP. Compared with RF alone, RF + LAAC remained associated with significantly lower risks of ischemic stroke/TIA (adjusted HR = 0.14, *P* = 0.005) and major bleeding (adjusted HR = 0.17, *P* = 0.020). No significant differences were observed in heart failure rehospitalization (adjusted HR = 1.42, *P* = 0.633), all-cause mortality (adjusted HR = 0.27, *P* = 0.152), or AF recurrence (adjusted HR = 0.83, *P* = 0.488).

## 4 Discussion

### 4.1 Major findings

This study investigated the safety and long-term prognosis of RF + LAAC procedure in the treatment of AF in patients complicated with HFpEF, in comparison with RF, and the major findings were as follows: (1) RF + LAAC demonstrated an acceptable safety profile in patients with AF and HFpEF, with no significant difference in postoperative complications compared to RF alone; (2) Compared to RF alone, RF + LAAC treatment did not increase the risk of HF rehospitalization in patients with AF and HFpEF, while significantly reducing ischemic stroke/TIA and major bleeding events.

### 4.2 Impact of RF + LAAC therapy on cardiac function in patients with AF and HFpEF

Previous studies have shown that catheter ablation can effectively improve LAD, LVEF, NYHA classification, and lower BNP levels in HFpEF patients (16). In addition, compared to drug therapy, catheter ablation can effectively reduce HF rehospitalization events in patients with AF and HFpEF (17).

However, under the circumstance of RF + LAAC treatment, the situation may differ. Currently, the impact of LAAC on cardiac function remains controversial. Some studies have found that LAAC can lead to functional and structural remodeling of the left atrium and left ventricle, which may further promote the progression of AF and HF (18). On the other hand, subanalysis of PRAGUE-17 study data found that although LAAC had no significant effect on NT-proBNP level, it might lead to more frequent HF hospitalization (19). What's more, the effect of LAAC on cardiac remodeling also appears complex. Studies have shown that echocardiography at 6 months post-LAAC indicated a significant increase in LAD, but during the 12- to 36-month follow-up period, LAD size gradually returned to preoperative levels (20).

Nevertheless, the potential negative impact of LAAC on cardiac function could be compensated or even reversed when combined with RF. This was first demonstrated in patients with AF and HFmrEF/HFrEF (7). Findings of this study further confirmed this perspective in HFpEF patients. In this study, no significant difference was observed between the RF + LAAC group and the RF group in HF rehospitalization events.

### 4.3 The preventive effect of RF + LAAC on embolism/bleeding events in patients with AF and HFpEF

For patients with AF combined with HF, existing randomized controlled studies did not show significant advantage of RF in reducing death, stroke, serious bleeding and cardiac arrest, compared with drug therapy (21). Therefore, after completing ablation, standardized anticoagulation therapy is still needed. It is important to emphasize that patients with AF and HFpEF have a higher risk of embolic events (22–24). However, studies have shown that in HF patients with persistent AF, the use of oral anticoagulants also increases the risk of bleeding (25).

The value of LAAC in reducing embolic events and drug-related bleeding events has been partially confirmed. Previous studies have shown that compared to warfarin, LAAC can significantly reduce hemorrhagic stroke and all-cause mortality events; compared to DOACs, LAAC demonstrates non-inferiority in major endpoints of embolic and bleeding events (26–28). What's more, the latest OPTION trial also revealed that in patients who had previously received RF, compared with anticoagulants, LAAC is associated with a significantly lower risk of bleeding events (29).

In this study, RF + LAAC treatment is associated with less ischemic stroke/TIA events in patients with AF and HFpEF compared to RF alone. This may be attributed to the higher risk of embolic events in patients with AF and HFpEF. The IMPRESSION LAAC study also provided a new perspective, taking the impact of device compression into consideration (30). This dual-center observational study suggested that the intentional usage of larger WATCHMAN FLX devices to obtain a device compression of >30% was associated with a lower rate of PDL. Although in our study we used WATCHMAN 2.5 device, the selection of device size could still be a contributor to the lower stroke risk in the RF + LAAC group.

Although LAA closure is biologically plausible for reducing thromboembolism originating from the LAA, stroke in patients with AF and HFpEF is multifactorial. Residual events may arise from non-LAA cardiac thrombi, atrial cardiomyopathy, aortic or carotid atherosclerosis, or cerebral small-vessel disease.

It should be emphasized that current AF guidelines recommend LAAC primarily for patients with contraindications to long-term oral anticoagulation or those at high risk of bleeding (9). Therefore, the lower rate of ischemic stroke/TIA observed in the RF + LAAC group should not be interpreted as evidence supporting routine replacement of oral anticoagulation in all patients undergoing AF ablation.

Regarding bleeding events during the follow-up period, the RF + LAAC group demonstrated a significantly lower bleeding risk compared to the RF group, which is coherent with current findings, suggesting a potential superiority of RF + LAAC.

### 4.4 Limitations

First, this study is a single-center, retrospective cohort study, which may introduce selection bias and information bias, limiting the generalizability of the results. The RF + LAAC cohort was derived from the LAACablation registry, in which only successful LAAC cases were enrolled, therefore the impact of success rate was not taken into consideration. Although this study used PSM to adjust for confounding factors, it cannot completely eliminate residual confounding, especially for unmeasurable potential confounders such as preference of both physician and patients, medical costs and economic status, antithrombolism adherence and lifestyles, which were also not systematically collected. This might lead to bias on clinical decision and outcome events. AF recurrence was assessed by intermittent ECG and Holter monitoring, which could result in an underestimated recurrence rate.

Due to the complex and highly heterogeneous pathophysiological mechanisms of HFpEF, this study did not further explore the potential impact of different HFpEF subtypes on the efficacy of RF + LAAC. The low number of ischemic stroke/TIA and major bleeding events resulted in wide confidence intervals; therefore, the precision and robustness of these estimates are limited. Although all of the ischemic/TIA events during the follow-up could be confirmed through imaging evidence and medical records, routine neuroimaging assessment or modified Rankin Scale data were not systematically available, causing potential underestimation of stroke risk in both groups. Additionally, this study primarily focused on major cardiovascular outcomes such as HF rehospitalization, stroke/TIA, and major bleeding events, but did not assess important prognostic indicators like quality of life (QoL), exercise tolerance.

## 5 Conclusion

In patients with AF and HFpEF, RF + LAAC was associated with lower risks of ischemic stroke/TIA and major bleeding events without an observed increase in heart failure rehospitalization compared with RF alone.

## Data Availability

The original contributions presented in the study are included in the article/Supplementary Material, further inquiries can be directed to the corresponding authors.
